# Aptamer‐functionalized field‐effect transistor biosensors for disease diagnosis and environmental monitoring

**DOI:** 10.1002/EXP.20210027

**Published:** 2023-05-11

**Authors:** Jingfeng Wang, Duo Chen, Wanting Huang, Nianjun Yang, Quan Yuan, Yanbing Yang

**Affiliations:** ^1^ College of Chemistry and Molecular Sciences, Institute of Molecular Medicine Renmin Hospital of Wuhan University, School of Microelectronics, Wuhan University Wuhan China; ^2^ Molecular Science and Biomedicine Laboratory (MBL), State Key Laboratory of Chemo/Biosensing and Chemometrics, College of Chemistry and Chemical Engineering Hunan University Changsha China; ^3^ Department of Chemistry, Insititute of Materials Research Hasselt University Hasselt Belgium

**Keywords:** aptamer, biosensor, disease diagnosis, environmental monitoring, field‐effect transistor

## Abstract

Nano‐biosensors that are composed of recognition molecules and nanomaterials have been extensively utilized in disease diagnosis, health management, and environmental monitoring. As a type of nano‐biosensors, molecular specificity field‐effect transistor (FET) biosensors with signal amplification capability exhibit prominent advantages including fast response speed, ease of miniaturization, and integration, promising their high sensitivity for molecules detection and identification. With intrinsic characteristics of high stability and structural tunability, aptamer has become one of the most commonly applied biological recognition units in the FET sensing fields. This review summarizes the recent progress of FET biosensors based on aptamer functionalized nanomaterials in medical diagnosis and environmental monitoring. The structure, sensing principles, preparation methods, and functionalization strategies of aptamer modified FET biosensors were comprehensively summarized. The relationship between structure and sensing performance of FET biosensors was reviewed. Furthermore, the challenges and future perspectives of FET biosensors were also discussed, so as to provide support for the future development of efficient healthcare management and environmental monitoring devices.

## Graphical abstract

Aptamer‐functionalized field‐effect transistor (FET) biosensors with merits of fast response speed, ease of miniaturization, and integration offer an attractive solution to disease diagnosis and environmental monitoring. This review is focusing on the development of aptamer‐functionalized FET biosensors from sensing mechanism to functionalization strategy to biological application level, combined with the latest research and in‐depth outlook, providing support for the future development of efficient medical management and environmental monitoring.

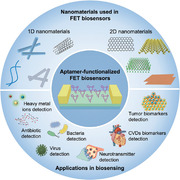

## 1 INTRODUCTION

Nano‐biosensors are emerging sensing devices that use nanomaterials as biosensing medium and utilize specific biorecognition elements to achieve biomolecules detection.^[^ ^1^ ^]^ Field‐effect transistor (FET) biosensors that were composed of source, drain, gate, and channel materials are promising as high sensitivity nano‐biosensors due to their intrinsic signal amplification capability.^[^ ^2^ ^]^ FET biosensors could generate an obvious and fast current change upon the contact of target biomolecules, enabling their capability for in situ biomolecules monitoring.^[^ ^3^ ^]^ Since there exists no charge transfer between biomolecules and channel materials, FET biosensors could well maintain the intrinsic properties of biomolecules, and thus benefit for efficient in vivo biosensing. A variety of one‐dimensional and two‐dimensional nanomaterials including carbon‐based nanomaterials,^[^ ^4^ ^]^ silicon nanowires (SiNWs),^[^ ^5^ ^]^ metal oxides,^[^ ^6^ ^]^ conductive polymers,^[^ ^7^ ^]^ transition metal sulfides^[^ ^8^ ^]^ and binary group III‐IV materials^[^ ^9^ ^]^ have been widely applied as channel materials of FET biosensors to achieve efficient detection of biomolecules. Additionally, the miniaturization characteristic of FET biosensors promises their capability as highly integrated devices for portable and point‐of‐care detection.^[^ ^10^ ^]^ Owing to their prominent characteristics, FET biosensors have been successfully applied in medical diagnosis and environmental monitoring.^[^ ^11^ ^]^

As a type of biorecognition element, aptamers have been extensively utilized in the FET biosensors owing to their unique advantages of structure tunability, high affinity, and stability.^[^ ^12^ ^]^ Since the successful development of the first batch of RNA aptamer in 1990, thousands of aptamers that could identify a series of targets including metal ions, organic molecules, peptides, proteins, viruses, bacteria, and cells have been explored.^[^ ^13^ ^]^ Additionally, aptamers could be conjugated on the FET device without compromising their molecular specificity. With the development of DNA nanotechnology, aptamer functionalized FET biosensors have been successfully applied in the detection of SARS‐CoV‐2,^[^ ^14^ ^]^ antibiotic,^[^ ^15^ ^]^ cancer biomarkers,^[^ ^16^ ^]^ heavy metal ions,^[^ ^17^ ^]^ and so forth. Moreover, aptamer functionalized FET biosensor could be easily fabricated into large‐scale biosensor arrays to satisfy the requirement of high‐throughput biosensing and molecular identification.

In this review, the recent progresses of aptamer‐functionalized FET biosensors for medical diagnosis and environmental monitoring were overviewed. First, the biosensor structure, working principle, and channel nanomaterials of FET biosensors were introduced. In the second part, the functionalization strategies of aptamer on the FET devices were briefly summarized. The application of aptamer‐functionalized FET biosensors in the fields of medical diagnosis and environmental monitoring was then comprehensively reviewed. Finally, the current challenges and future opportunities regarding the design of miniature and integrated high‐performance aptamer‐functionalized FET biosensors were discussed. Overall, this review offers a strategy for the preparation of aptamer functionalized FET biosensors, and further promotes the application of FET biosensors in massive health data monitoring.

## 2 FET BIOSENSORS

### 2.1 Working principles of FET biosensors

**FIGURE 1:**
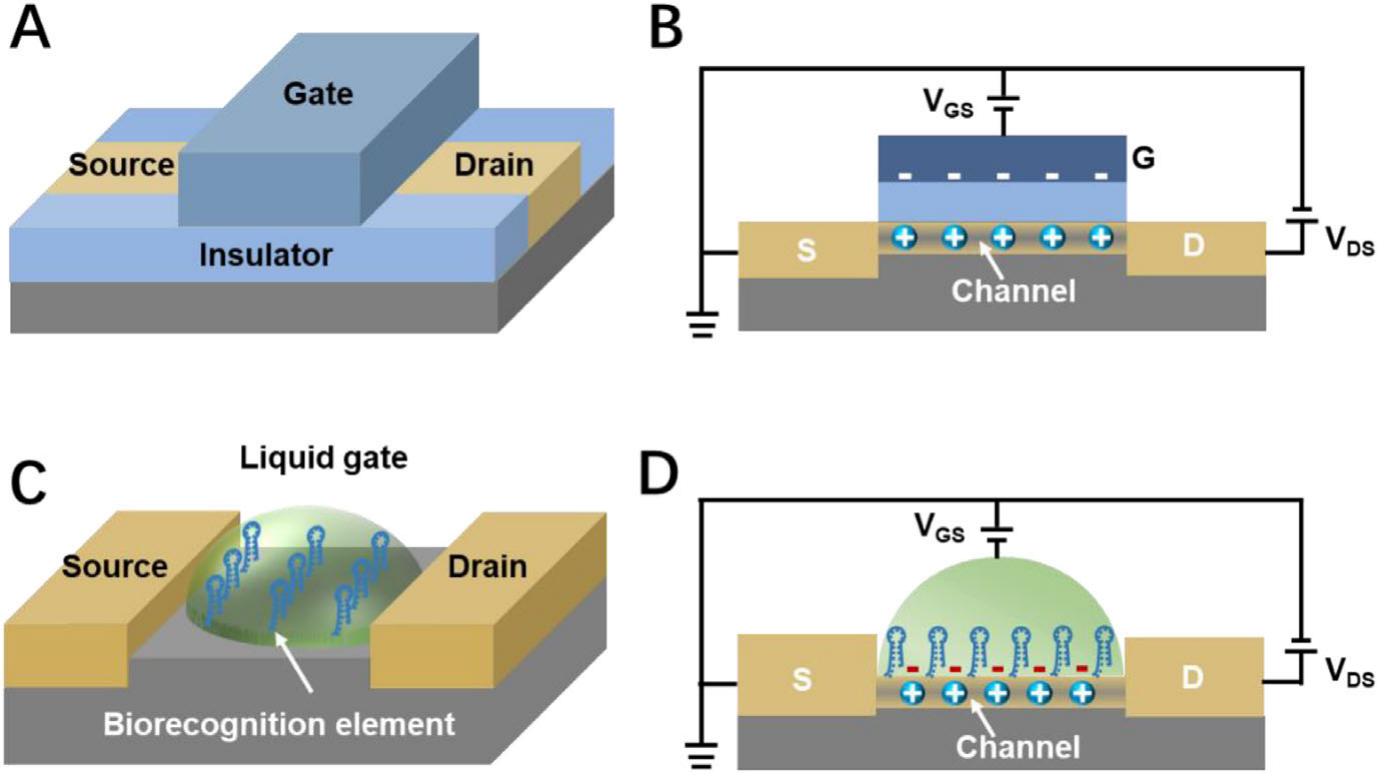
Working principles of field‐effect transistor (FET) biosensors. A) Schematic illustration of a typical FET device. B) Working principle of a p‐channel type FET. C) Schematic illustration of a liquid‐gate FET biosensor. D) Sensing mechanism of a p‐channel type FET biosensor.

FET is a semiconductor device that is composed of channel materials, source (S), drain (D), and gate (G) electrodes (Figure 1A).^[^ ^18^ ^]^ The FET works by modulating the conductance between the insulating layer and the semiconductor channel layer through the G electric field. A current between S and D electrodes (*I*_ds_) forms under the action of source and drain voltages (*V*_ds_) (Figure 1B). According to the characteristics of prime charge carriers, FET devices are mainly divided into two categories: n‐channel type (electrons are majority charge carriers) and p‐channel type devices (holes are majority charge carriers).^[^ ^19^ ^]^ The response current of p‐channel type FET current will increase due to electron aggregation when the target molecule is negatively charged.^[^ ^20^ ^]^ On the contrary, the device response current shows a decrease when a positively charged molecule is conjugated on the device.^[^ ^21^ ^]^ The opposite phenomenon occurs for n‐channel type FET devices.^[^ ^22^ ^]^

To achieve selective biosensing, the FET devices should be functionalized with biorecognition elements such as aptamer,^[^ ^23^ ^]^ antibody,^[^ ^24^ ^]^ DNA,^[^ ^25^ ^]^ or enzyme,^[^ ^26^ ^]^ and so forth. (Figure 1C). During the biosensing process, the binding of biorecognition element with target molecules would cause the consumption or accumulation of charge in the channel materials and generate a recordable current signal (Figure 1D). The variation of charge density could seem as electrostatic gating or Schottky barrier modulation on the FET biosensor.^[^ ^27^ ^]^ Similarly, taking p‐channel type FET biosensor as an example, the binding of positively charged target is equivalent to the positive gating of FET biosensor and would cause the decrease of current.^[^ ^28^ ^]^ When the biorecognition unit binds a negatively charged target, the response current of p‐channel FET biosensor will increases owing to charge accumulation.^[^ ^29^ ^]^

### 2.2 Channel nanomaterials used in FET biosensors

**FIGURE 2:**
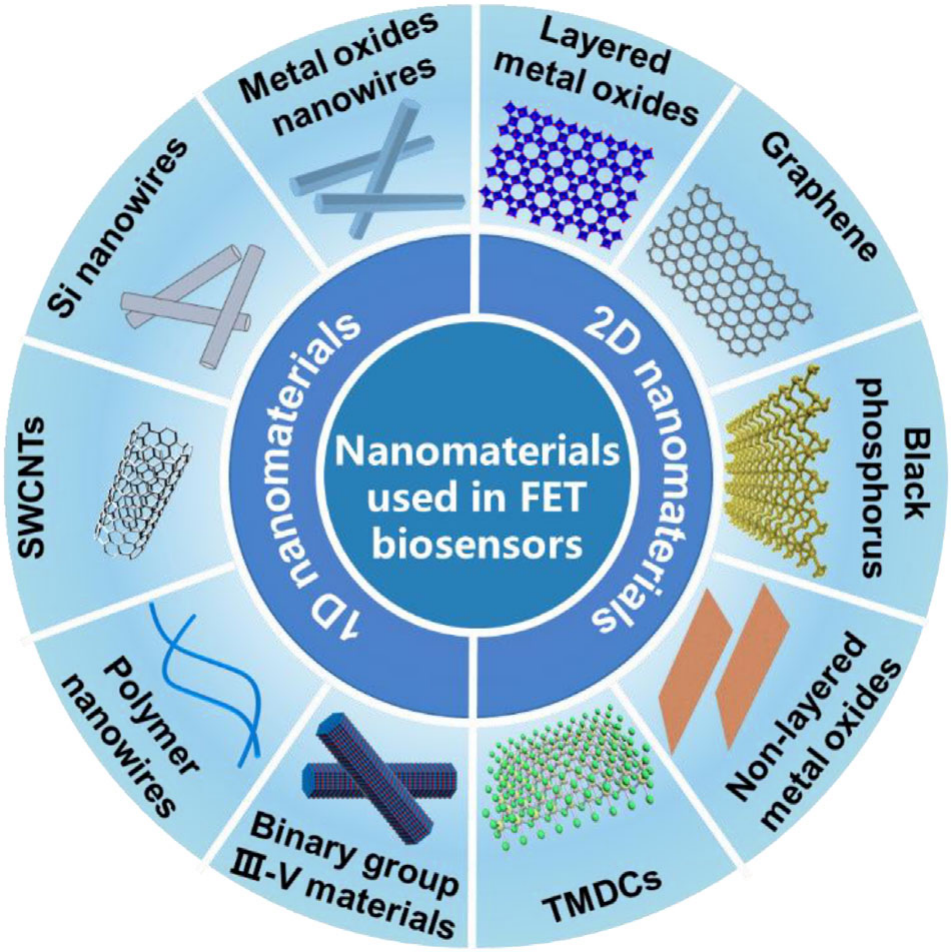
Schematic illustration showing the typical 1D and 2D nanomaterials used in field‐effect transistor (FET) biosensors. TMDCs, transition metal dichalcogenides.

The structure and physical performance of channel materials directly determine the electrical performance of FET device and the sensitivity of biosensor.^[^ ^30^ ^]^ The design and construction of channel materials with high surface to volume ratio, excellent electrical performance, and biocompatibility are necessary for biosensing applications.^[^ ^31^ ^]^ Figure 2 summarizes the typical nanomaterials used in FET biosensors. For one‐dimensional (1D) semiconductors, SiNWs, carbon nanotubes (CNTs), metal oxides nanowires (NWs) or nanoribbons (NBs), conductive polymer nanowires and binary group III‐V materials with high current on/off ratio (∼ 10^6^), high carrier mobility (∼ 8.0 cm^2^ V^−1^ s^−1^) and large surface‐to‐volume ratio are ideal for constructing highly sensitive FET biosensors.^[^ ^32^ ^]^ Two‐dimensional (2D) materials such as graphene, transition metal oxides, black phosphorus (BP), transition metal dichalcogenides (TMDCs), and non‐layered metal oxides exhibit prominent characteristics such as large surface area, high current on/off ratio (up to 1000) and carrier mobility, excellent mechanical strength, and ease to be regulation, and they have been extensively utilized for fabricating high performance FET biosensors.^[^ ^33^ ^]^ 2D materials play an important role in the biosensors because of their many unique properties such as large surface area, which is more amenable to microfabrication techniques and to large‐area biofunctionalization than 1D materials. The following section would introduce the typical nanomaterials used in FET biosensors.

SiNWs are the most commonly used 1D channel materials in FET biosensors, and could be obtained through vapor deposition, thermal evaporation, and electrochemical methods.^[^ ^34^ ^]^ Unlike conventional planar silicon‐based FETs, SiNWs exhibit improved sensitivity due to reduced dimensionality and large specific surface area.^[^ ^35^ ^]^ SiNWs FET can bind biological targets through direct or indirect aptamer modification, and convert biological/chemical charge information into electrical signals.^[^ ^36^ ^]^ SiNWs‐based FETs biosensors have so far been successfully utilized in real‐time, label‐free detection of a wide range of biomolecules such as pH, nucleic acids, proteins, and action potentials in physiological solutions with high ionic strength.^[^ ^37^ ^]^ With large specific surface area and high carrier mobility, 1D semiconductor CNTs have also been used as channel materials for constructing FET biosensors.^[^ ^38^ ^]^ CNTs are usually prepared by chemical vapor deposition. The high carrier mobility allows for the fast transport of electrons, thus guaranteeing its excellent biosensing properties.^[^ ^39^ ^]^ The surface of CNTs could be easily functionalized with different chemical groups and biological receptors through covalent or non‐covalent strategies.^[^ ^40^ ^]^ Owing to their high sensitivity, stability, fast response, and wide detection range, FET biosensors based on CNTs have been widely used in the development of various functionalized biosensors such as glucose biosensors, hydrogen peroxide biosensors, metal ions biosensors, and immunosensors.^[^ ^41^ ^]^

Mechanical exfoliation and chemical vapor deposition are the main methods to prepare 2D materials. Graphene materials including graphene, graphene oxide (GO), and reduced graphene oxide (rGO) are widely used as channel materials due to their large specific surface area, excellent biocompatibility, and extraordinary electron transport properties.^[^ ^42^ ^]^ For biosensing applications, the graphene plane could be modified with immobilized bioreceptors (e.g. aptamer, antibodies, or single strand DNA probes) through direct adsorption or via a linker molecule.^[^ ^43^ ^]^ A variety of graphene‐based FET biosensors have been constructed for sensitively detecting enormous analytes including DNA, micro ribonucleic acid (miRNA), proteins, pathogenic virus, glucose, and neurotransmitters.^[^ ^44^ ^]^ In addition, TMDs are ideal candidates for developing next‐generation high performance FET biosensors due to their unique features, including a direct band gap and atomic thickness along with their excellent mechanical, optical, and electrical properties.^[^ ^45^ ^]^ As a typical representative of TMDs, molybdenum disulfide (MoS_2_) with graphene‐like layered structure has broad application prospects in biosensors due to its large specific surface area, high electrochemical activity, and specific affinity with biomolecules.^[^ ^46^ ^]^ MoS_2_ FET biosensors have been successfully applied in the detection of various biomolecules, including DNA, protein, glucose, and so forth.^[^ ^47^ ^]^

## 3 APTAMER FUNCTIONALIZED FET BIOSENSORS

### 3.1 Advantages of aptamer in biosensing

Aptamers are single‐stranded nucleic acid molecules obtained through systematic evolution of ligands by exponential enrichment (SELEX).^[^ ^48^ ^]^ As the analogs of antibodies, aptamers could recognize a variety of target analytes such as ions,^[^ ^49^ ^]^ small molecules,^[^ ^50^ ^]^ proteins,^[^ ^51^ ^]^ cells,^[^ ^52^ ^]^ pathogens,^[^ ^53^ ^]^ and even tissues^[^ ^54^ ^]^ with high selectivity. The molecular recognition process between aptamer and targets is similar to antibody‐antigen interactions, they are mainly directed by hydrogen bonds, hydrophobic interactions, and van der Waals forces.^[^ ^55^ ^]^ Based on the specific affinity of aptamer and target, aptamer‐functionalized FET biosensors can selectively recognize biological targets and convert target information into a prominent electrical signal to achieve efficient detection. With a small volume and compact structure, aptamer could be densely immobilized on the sensing interface, thereby improving the target binding efficiency.^[^ ^56^ ^]^ Additionally, the compact structure of aptamer narrows the distance between channel materials and biological target, allowing for the recognition molecule‐target interaction located in the range of Debye length (*λ*_D_) where the electrical signal can be efficiently detected. It is noted that this enhanced interaction is necessary to overcome the decrease in sensing sensitivity caused by Debye screening effect at high ionic strength in complex biological environment.^[^ ^57^ ^]^ Compared with antibodies, aptamers could be easily modified and synthesized.^[^ ^58^ ^]^ At the same time, the synthesis process of aptamers is a chemical process rather than a biological process, so it avoids the problem of biological contamination and reduces the variability between different production batches.^[^ ^59^ ^]^ Aptamers also exhibit excellent stability and can be stored for a long time at room temperature. Upon unfolding, aptamers can be refolded to the functional state after a simple annealing treatment.^[^ ^60^ ^]^ More importantly, aptamers could be chemically modified with different functional groups to satisfy different requirements and applications.^[^ ^61^, ^62^, ^63^, ^64^ ^]^ Based on these prominent characteristics, aptamers could provide ultrahigh sensitivity and specificity, and they have become a powerful tool for advanced biological and biomedical applications.^[^ ^65^, ^66^, ^67^, ^68^, ^69^ ^]^

### 3.2 Aptamer functionalization strategies

The aptamer functionalization strategies are mainly divided into covalent and non‐covalent strategies.^[^ ^70^ ^]^ The non‐covalent functionalization strategy is based on the physical process of π–π stacking and electrostatic interaction, and the covalent functionalization strategy relies on the chemical functionalization.

#### 3.2.1 Non‐covalent strategies

**FIGURE 3:**
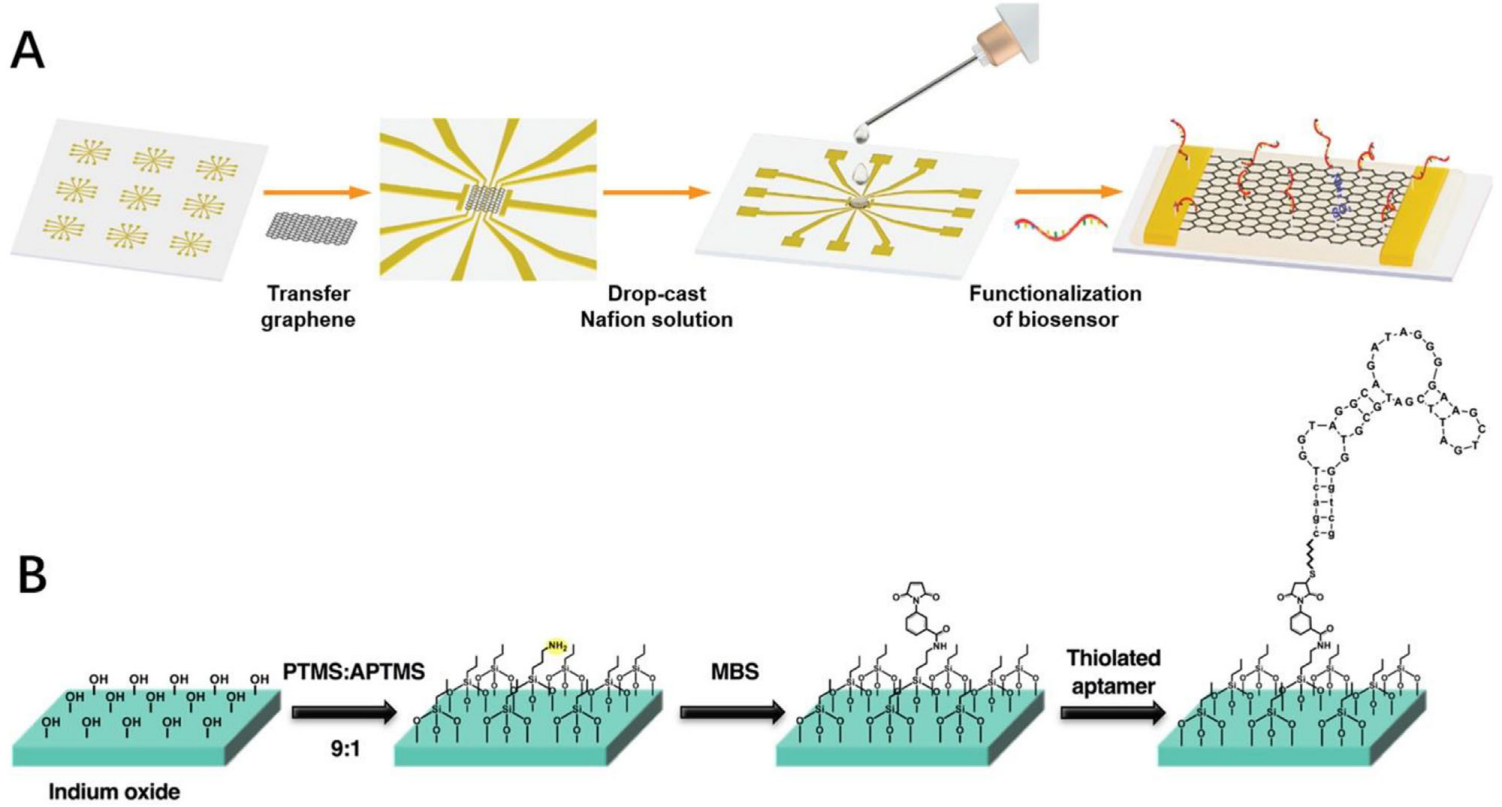
Aptamer functionalization strategies. A) Modification of aptamer on the field‐effect transistor (FET) device through electrostatic interaction. Reproduced with permission.^[^ ^65^ ^]^ Copyright 2021, John Wiley & Sons. B) Schematic of aptamer modification based on covalent bonds strategy. Reproduced with permission.^[^ ^77^ ^]^ Copyright 2018, The American Association for the Advancement of Science. MBS, 3‐maleimidobenzoic acid *N*‐hydroxysuccinimide ester.

As one of the most commonly used non‐covalent functionalization strategies, electrostatic interaction mainly relies on the electrostatic adsorption between aptamer and channel materials.^[^ ^71^ ^]^ Pan et al. developed a flexible and reproducible FET biosensor by non‐covalent functionalization of aptamers onto the surface of graphene‐Nafion composite film (Figure 3A).^[^ ^72^ ^]^ The graphene‐Nafion composite film with negatively charged sulfonic acid groups was interacted with positively charged amine groups of INF‐γ‐specific aptamer through electrostatic interaction. The non‐covalent modification strategy is relatively mild and avoids the requirement of complicated operation procedures, reducing the possibility of aptamer denaturation and maintaining the electrical characteristics of channel materials. However, the relatively weak binding force between aptamer and channel materials may induce the dissociation of aptamer and reduce the detection reproducibility of FET biosensors.

#### 3.2.2 Covalent modification strategies

Compared with non‐covalent modification, covalent modification could provide enhanced binding force between aptamer and FET device, resulting in improved detection reproducibility and sensing stability. The commonly used covalent functionalization strategies rely first on the functionalization treatments such as amination,^[^ ^73^ ^]^ carboxylation,^[^ ^74^ ^]^ hydroxylation,^[^ ^75^ ^]^ or gold deposition^[^ ^76^ ^]^ on the FET channel, and then fix one end of the linker molecule such as 3‐maleimidobenzoic acid *N*‐hydroxysuccinimide ester (MBS),^[^ ^77^ ^]^ 1‐pyrenecarboxylic acid,^[^ ^78^ ^]^ 1‐pyrenebutyric acid succinimidyl ester (PBASE),^[^ ^79^ ^]^ glutaraldehyde,^[^ ^80^ ^]^ and so forth. on the functionalized substrate surface through covalent bonds^[^ ^81^ ^]^ or π–π stacking.^[^ ^82^ ^]^ Finally, the other end of linker molecule is conjugated with the functionalized aptamers through covalent bonds to complete the biological recognition unit immobilization process.^[^ ^83^ ^]^ A typical example is Anne et al. fabricated a series of FET biosensors using chemical modification methods to immobilize aptamers on In_2_O_3_ (Figure 3B).^[^ ^84^ ^]^ Specifically, the substrate was first subjected to an amination treatment by vapor‐phase deposition of (3‐aminopropyl)trimethoxysilane and trimethoxy(propyl)silane onto the In_2_O_3_ surface. The linker molecule MBS is then immobilized on the substrate through the formation of amide bond between *N*‐hydroxysuccinimide and the amino group. Finally, the maleimide group at the MBS reacts with the thiol group of thiolated DNA aptamer to form a thioether bond to complete the immobilization of aptamer on the substrate. The covalent modification strategy could immobilize aptamer on the surface of channel materials firmly and stably, even allowing for orientation‐specific immobilization, endowing the device with good stability. However, covalent modification strategies require case‐by‐case sequence design, multi‐step synthesis, and purification. Any changes in the aptamer stem‐loop structure during this time may affect the conformational stability of aptamer, compromising binding affinity or detection specificity. It is recognized that appropriate modification methods could be chosen to functionalize FET biosensors depending on the properties of channel materials and biorecognition elements, thereby satisfying different demands of FET biosensors.

## 4 APPLICATION OF APTAMER FUNCTIONALIZED FET BIOSENSORS

Aptamer functionalized FET biosensors can readily achieve high sensitivity and specificity due to the excellent addressability and spatial tunability of aptamers.^[^ ^85^ ^]^ Up to now, aptamer functionalized FET biosensing devices play vital roles in numerous fields. In the following sections, we will comprehensively discuss their applications in medical diagnosis and environmental monitoring.

### 4.1 Application in disease diagnosis

Early and accurate diagnosis of diseases could improve the chance of successful treatment.^[^ ^86^ ^]^ Biomarkers are molecules that are closely related to the occurrence and development of diseases.^[^ ^87^ ^]^ The early diagnosis, monitoring, and prognosis of diseases can be achieved by analyzing the corresponding biomarkers information.^[^ ^88^ ^]^ However, the low concentrations of biomarkers at the early stages of diseases can easily lead to missed diagnosis.^[^ ^89^ ^]^ Additionally, the existence of enormous interference molecules such as non‐specific proteins, nucleic acids, and ions in the biological environment would cause serious non‐specific adsorption and make the detection of trace biomarkers more difficult.^[^ ^90^ ^]^ Aptamer functionalized FET biosensors exhibit high sensitivity, coupled with signal amplification capability based on gate‐controlling potential of FET device, it is expected to achieve high sensitivity and specificity detection of low‐concentration or even single‐molecule level biomarkers in complex biological environment and achieve effective disease diagnosis.^[^ ^91^ ^]^ The aforementioned advantages have led to a surge in research activity on aptamer‐functionalized FET biosensors for disease diagnosis and monitoring by analyzing biomarkers in body fluids in vivo and in vitro. Table 1 summarizes the recent advances of aptamer functionalized FET biosensors in the fields of disease diagnosis.

**TABLE 1:** Representative summary of aptamer functionalized FET biosensors in the fields of disease diagnosis.

Aptamer functionalized FET biosensors	Disease	Biomarkers	Linear range	Detection limit	Ref.
CPPyNW/graphene FET	Tumor	Hepatitis B surface antigen	10^‒18^–10^‒7^ M	10^‒18^ M	[85b]
GFET	Tumor	HER2	6 × 10^‒16^–6 × 10^‒11^ M	6 × 10^‒16^ M	[92]
(AuNPs)/rGO FET	Tumor	HepG2 MVs	6 × 10^5^–6 × 10^9^ particles mL^−1^	8.4 × 10^4^ particles mL^−1^	[93]
SiNW FET	Tumor	PSA	3 × 10^‒13^–3 × 10^‒8^ M	3 × 10^‒13^ M	[94]
MWCNTs/rGO FET	Tumor	CA125	10^−9^–1 U mL^−1^	5 × 10^−10^ U mL^−1^	[95]
GFET	Tumor	Vascular endothelial growth factor 165	10^−11^ ‐ 8 × 10^‒7^ g mL^−1^	3.24 × 10^−12^ g mL^‒1^	[96]
Graphene/Nafion FET	Tumor	IFN‐γ	1.5 × 10^−11^–2.5 × 10^−7^ M	7.4 × 10^−13^ M	[72]
GFET	Tumor	Tumor necrosis factor‐α	8 × 10^−12^–1.25 × 10^−7^ M	3.4 × 10^−13^ M	[97]
GFET	Tumor	Tumor necrosis factor‐α	5 × 10^−11^–10^−7^ M	5 × 10^−12^ M	[83a]
GFET	Tumor	Tumor necrosis factor‐α	2 × 10^−10^–2 × 10^−17^ M	2.6 × 10^−11^ M	[98]
C‐PPy MNT FET	Tumor	Carcinoembryonic antigen	10^−16^–10^−11^ g mL^−1^	10^−15^ g mL^−1^	[99]
CNT FET	Tumor	Interleukin‐6	10^−12^–10^−8^ g mL^−1^	10^−12^ g mL^−1^	[100]
GFET	Tumor	Interleukin‐6	10^−11^–10^−7^ M	10^−11^ M	[101]
GFET	Tumor	Interleukin‐6	1.5 × 10^−12^–10^−7^ M	1.39 × 10^−13^ M	[102]
SiNW FET	CVDs	cTnI	10^−10^–10^−16^ g mL^−1^	10^−10^ g mL^−1^	[103]
PEG/graphene FET	CVDs	cTnI	10^−11^–5 × 10^−10^ g mL^−1^	3.34 × 10^−12^ g mL^−1^	[51]
AlGaN/GaN FET	CVDs	cTnI	6 × 10^−12^–1.48 × 10^−7^ g mL^−1^	6 × 10^−12^ g mL^−1^	[104]
SWCNT/Au‐Co_3_O_4_ FET	CVDs	cTnI	10^−7^–10^−5^ g mL^−1^	10^−7^ g mL^−1^	[105]
GFET	CVDs	Thrombin	10^−15^–10^−8^ M	10^−15^ M	[106]
GFET	CVDs	Thrombin	10^−12^–10^−6^ M	2.6 × 10^−12^ M	[107]
AlGaN/GaN FET	CVDs	CRP	2.6 × 10^−11^–2.6 × 10^−6^ M	2.6 × 10^−11^ M	[108]
AlGaN/GaN FET	CVDs	CRP, NT‐proBNP, cTnI, fibrinogen	10^−7^–5 × 10^−5^ g mL^−1^, 5 × 10^−11^–10^−8^ g mL^−1^, 10^−12^–10^−8^ g mL^−1^, 10^−4^–5 × 10^−3^ g mL^−1^	1.4 × 10^−7^ g mL^−1^, 8.32 × 10^−13^ g mL^−1^, 3.94 × 10^−13^ g mL^−1^, 2.02 × 10^−4^ g mL^−1^	[109]
PEDOT: PSS OECT	CVDs	Epinephrine	9 × 10^−11^–9 × 10^−7^ M	9 × 10^−11^ M	[110]
PEDOT: PSS OECT	Neurological diseases	ATP	10^−5^–10^−6^ M	10^−11^ M	[111]
In_2_O_3_ FET	Neurological diseases	Serotonin	10^−14^–10^−4^ M	10^−14^ M	[112]
Polyacrylonitrile/PEDOT OECT	Neurological diseases	Serotonin	10^−14^–10^−7^ M	10^−14^ M	[113]
GFET	Neurological diseases	Serotonin, DA	10^−11^–10^−4^ M, 10^−9^–10^−4^ M	10^−11^ M, 10^−11^ M	[114]
In_2_O_3_ FET	Neurological diseases	Serotonin, DA	10^−14^–10^−6^ M, 10^−14^–10^−6^ M	10^−14^ M, 10^−14^ M	[115]
In_2_O_3_ FET	Neurological diseases	DA	10^−11^–10^−7^ M	10^−11^ M	[116]
OECT	Neurological diseases	DA	5 × 10^−15^–10^−9^ M	5 × 10^−16^ M	[117]
SiNW FET	Neurological diseases	DA	10^−11^–10^−8^ M	10^−11^ M	[118]
SiNW FET	Neurological diseases	Amyloid beta‐40	10^−13^–10^−5^ g mL^‒1^	2 × 10^−15^ M	[36]
CNT FET	Neurological diseases	Amyloid‐β 40, amyloid‐β 42	10^−15^–10^−11^ M	5.5 × 10^−17^ M, 4.5 × 10^−17^ M	[119]
SiNW FET	Neurological diseases	Amyloid beta 1–42	10^−13^–10^−10^ g mL^−1^	10^−13^ g mL^‒1^	[120]
SiNW FET	Neurological diseases	K^+^	10^−9^–10^−6^ M	10^−9^ M	[121]
GFET	Pathogen	SARS‐CoV2 RNA	10^2^–5 × 10^5^ copies mL^−1^	10 copies mL^−1^	[10]
Si/Al FET	Pathogen	SARS‐CoV2 spike protein	10^−13^–10^−11^ M	10^−13^ M	[122]
Al_2_O_3_ FET	Pathogen	H_5_N_1_	10^−11^–10^−8^ M	5.9 × 10^−12^ M	[123]
MWCNTs FET	Pathogen	HIV‐1 Tat	6 × 10^−10^–10^−6^ M	6 × 10^−10^ M	[124]
AlGaN/GaN FET	Pathogen	HIV‐1 RT	10^−15^–10^−11^ M	10^−15^ M	[125]
GFET	Pathogen	*E. coli*	10^2^–10^6^ CFU mL^‒1^	10^2^ CFU mL^‒1^	[126]
SWCNT FET	Pathogen	*E. coli*	0.2–10^3^ CFU mL^−1^	0.2 CFU mL^−1^	[127]
rGO FET	Pathogen	Plasmodium falciparum lactate dehydrogenase	7.8 × 10^−16^–10^−7^ M	7.8 × 10^−16^ M	[128]
GFET	Diabetes	Anti‐diuretic hormone	10^−17^–10^−12^ g mL^−1^	3.55 × 10^−18^ g mL^−1^	[129]
PEG: SiNW FET	Diabetes	Glycated hemoglobin A1c	3 × 10^−9^–6 × 10^−7^ M	3 × 10^−9^ M	[130]
rGO FET	Diabetes	Retinol binding protein 4	3 × 10^−10^–3 × 10^−8^ M	2 × 10^−9^ g mL^‒1^	[131]
GFET	Diabetes	Insulin	10^−10^–10^−6^ M	3.5 × 10^−11^ M	[132]

Abbreviations: CRP, C‐reactive protein; CVDs, cardiovascular diseases; FET, field‐effect transistor; GFET, graphene‐based FET; MWCNT, multi‐walled carbon nanotubes; OECT, organic electrochemical transistor; PEDOT: PSS, poly(3,4‐ethylenedioxythiophene)‐doped poly(styrenesulfonate); SiNW, silicon nanowire.

#### 4.1.1 Tumor‐related biomarkers detection

**FIGURE 4:**
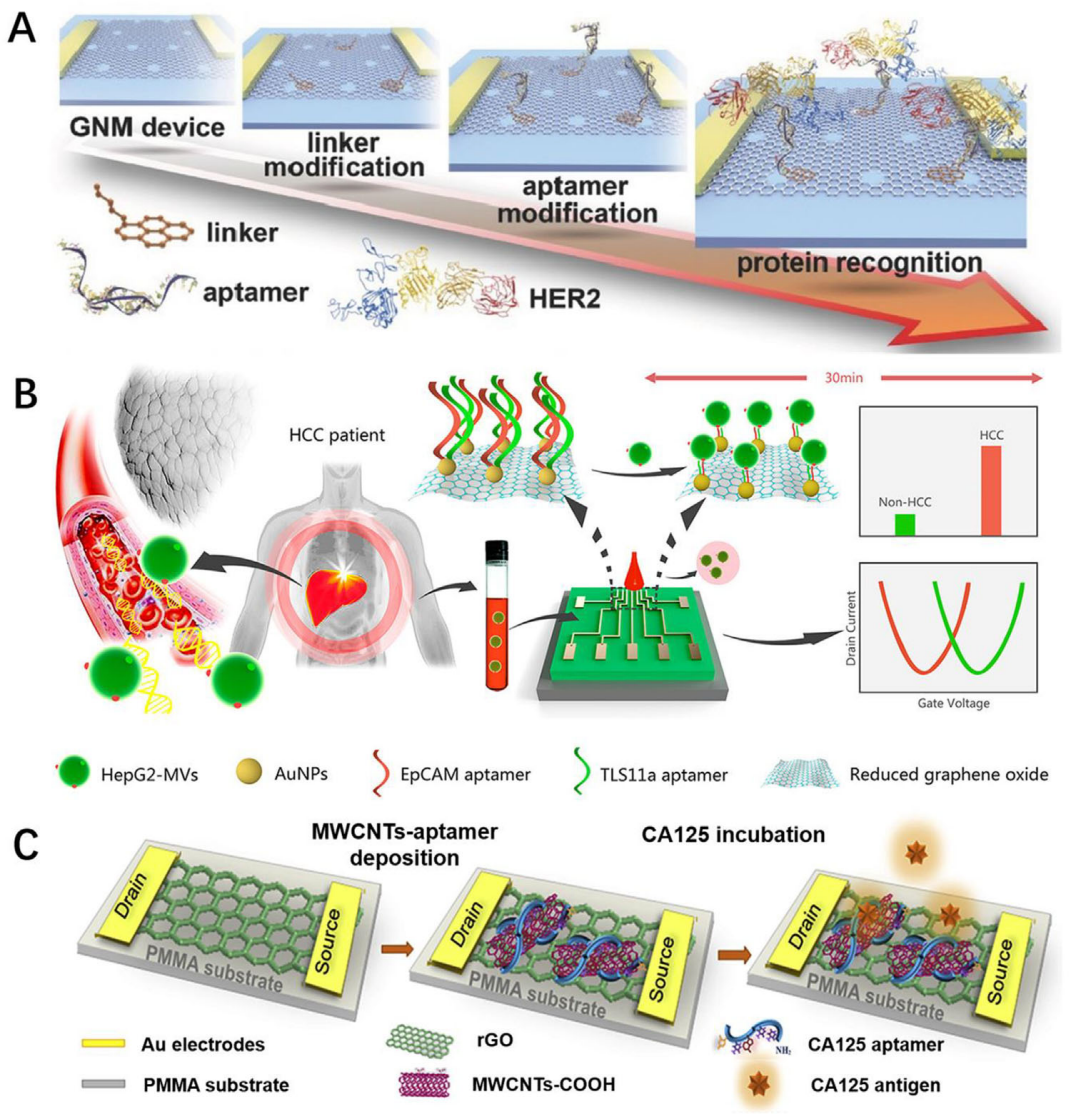
Tumor‐related biomarkers detection. A) Schematic of the fabrication process of HER2‐specific aptamer modified graphene nanomesh (GNM) field‐effect transistor (FET) biosensor. Reproduced with permission.^[^ ^92^ ^]^ Copyright 2017, John Wiley & Sons. B) Schematic representation of the electrical detection of HepG2‐MVs from blood samples using the dual‐aptamer modified AuNPs/rGO FET biosensor. Reproduced with permission.^[^ ^93^ ^]^ Copyright 2020, American Chemical Society. C) Construction of carboxylated multi‐walled carbon nanotubes (MWCNTs)/reduced graphene oxide FET aptasensor for label‐free detection of CA125. Reproduced with permission.^[^ ^95^ ^]^ Copyright 2018, Elsevier. HCC, hepatocellular carcinoma.

Breast cancer causes approximately 23% of cancer cases worldwide whose effective detection plays a crucial role in the disease early diagnosis and target treatment.^[^ ^133^ ^]^ Human epidermal growth factor receptor 2 (HER2) is a transmembrane protein overexpressed on the surface of breast cancer cells, and it has been comprehensively used as an important diagnosis indicator for breast cancer.^[^ ^134^ ^]^ Our group constructed a highly sensitive aptamer functionalized graphene nanomesh (GNM) biosensor for high sensitivity and selectivity detection of HER2 (Figure 4A).^[^ ^92^ ^]^ The GNM with a neck width of < 3 nm and high on/off ratio of 1000 promise high sensitivity for HER2 detection. The aptamer functionalization was then achieved by the conjugation of amino‐modified HER2‐specific aptamer with 1‐pyrenebutyric acid succinimidyl ester linker molecules on the GNM plane through the formation of amide bonds. The interaction between positively charged HER2 protein with negatively charged aptamers reduces the hole carrier density in the GNM channel, leading to a drop in current of GNM biosensor. The high sensitivity aptamer functionalized GNM biosensor achieves an ultralow detection limit of 0.6 × 10^‒15^ M for HER2.

Hepatocellular carcinoma (HCC) is the second most common cause of cancer death worldwide.^[^ ^135^ ^]^ Specific detection of HepG2 cell‐derived cancerous microvesicles (MVs) could reflect the expression level of disease‐related molecules on maternal cancer cells and be used to diagnose and monitor HCC.^[^ ^136^ ^]^ Zhang et al. designed a gold nanoparticles (AuNPs)‐deposited, dual‐aptamer modified rGO FET biosensor for the specific and sensitive quantification of HepG2 MVs (Figure 4B).^[^ ^93^ ^]^ The thiolated TLS11a aptamers and EpCAM aptamers are simultaneously functionalized onto AuNPs‐deposited FET biosensors via Au─S bonds. The detection principle relies on the specific recognition between HepG2‐MVs and the dual‐aptamer. As the negatively charged HepG2‐MVs is specifically captured by dual‐aptamer and accumulates on rGO surface, continuous n‐doping occurs in rGO channel, which induces a leftward shift of the dirac point. The uniquely designed dual‐aptamer‐based biosensing interface increases the sensitivity of HepG2‐MVs detection by at least 2 orders of magnitude compared to single aptamer modified devices, and the detection limit reaches as low as 84 particles μL^−1^.

Prostate specific antigen (PSA) is a clinically relevant tumor biomarker that has been widely used in the early diagnosis of prostate cancer.^[^ ^137^ ^]^ Rani et al. integrated a p‐type SiNWs‐FET array with a microfluidic system to construct a label‐free detection device for PSA.^[^ ^94^ ^]^ The SiNWs sensing channel was first covered with a layer of 3‐glycidoxypropyltrimethoxysiloxane (GPTMS) to create a hydroxyl‐terminated surface, and then connected aptamer on the sensing surface through the formation of amide bond. The thickness of the PSA‐specific aptamer with 32 nucleotides is about 1.2–1.8 nm, which shortens the distance between the PSA and the channel surface, enhances the surface field effect and is beneficial to improve the sensitivity of the SiNWs‐FET. The binding between PSA and aptamer results in an increase in the positively charged molecules on the surface of FET biosensor and further a significant change in the threshold voltage, which is proportional to the concentration of PSA. The integrated device exhibits a wide linear detection range of 3 pM–30 nM and a low LOD of 0.05 ng mL^−1^, which is much lower than the clinically determined PSA level of <4 ng mL^−1^.

Ovarian cancer is one of the deadliest gynecological malignancies worldwide, and the early diagnosis and treatment will greatly improve the clinical prognosis of patients.^[^ ^138^ ^]^ CA125 is a ovarian cancer relevant biomarker that is recommended for clinical use in the early diagnosis of ovarian cancer of high‐risk women.^[^ ^139^ ^]^ Salimi et al. demonstrated a flexible rGO‐based aptasensor for detecting CA125. CA125 specific aptamer was immobilized on the surface of carboxyl multi‐walled carbon nanotubes (MWCNTs) by *N*‐hydroxysuccinimide and 1‐ethyl‐3‐(3‐dimethylaminopropyl) carbodiimide hydrochloride chemistry (Figure 4C).^[^ ^95^ ^]^ Subsequently, CA125 specific aptamer wrapped MWCNTs were stacked on the rGO surface through π–π interaction to construct a flexible FET aptasensor. The binding of negatively charged CA125 to the FET induces the depletion of negative charge carriers in the rGO channel layer. The strong field‐induced response of the aptasensor resulted in a decrease in *I*_ds_ with increasing CA125 concentration, and had a recognizable electrical signal response at a low concentration of 5.0 × 10^−10^ U mL^‒1^. The highly sensitive, selective, and stable biosensing device realizes the detection of CA125 in human serum samples of ovarian cancer patients. Meanwhile, the rGO‐based aptasensor exhibits reproducible bending flexibility on PMMA substrates, providing a feasible technical practice for the construction of portable, wearable and even implantable biosensors.

#### 4.1.2 Cardiovascular disease relevant biomarkers detection

**FIGURE 5:**
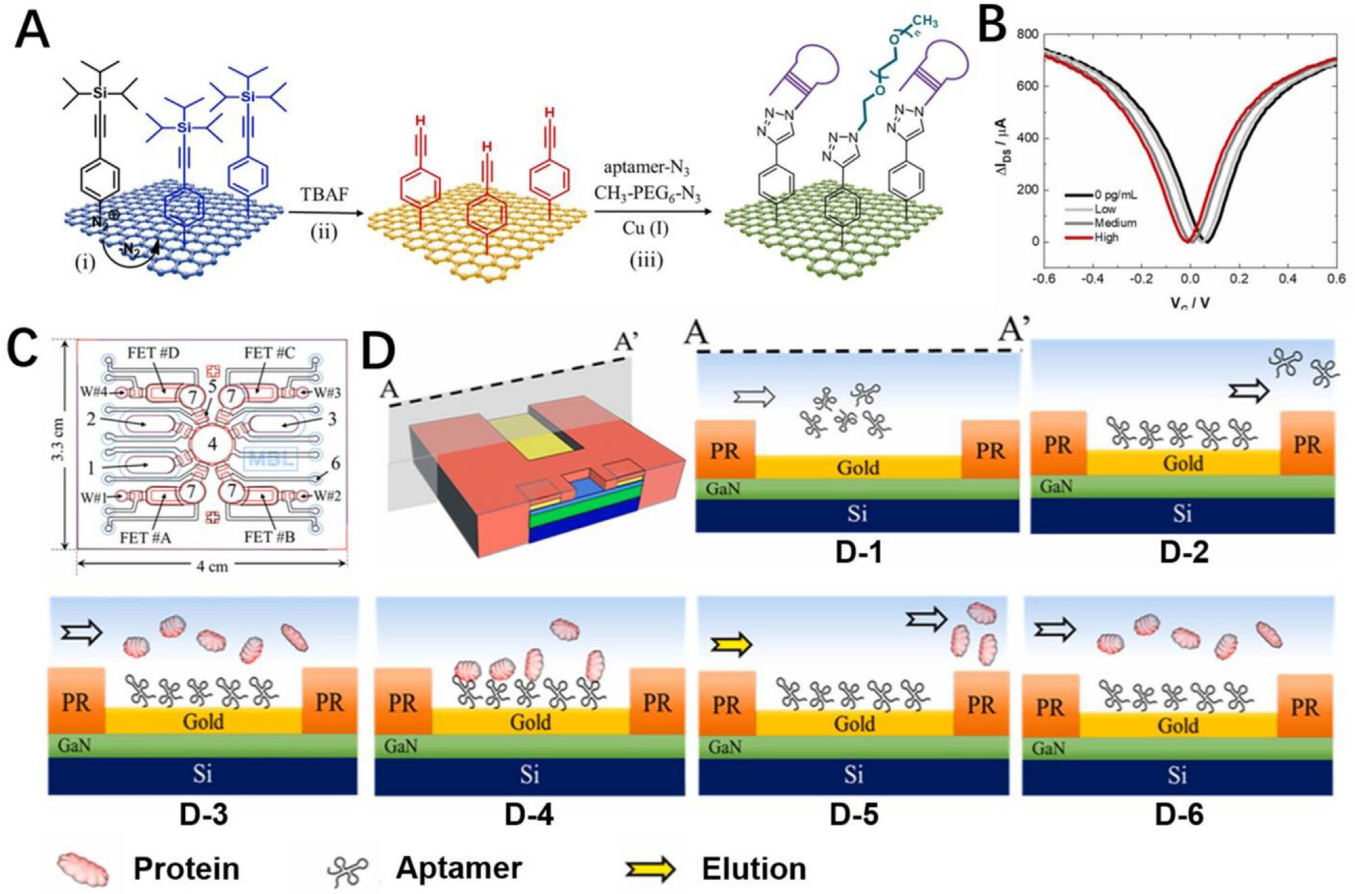
Cardiovascular disease relevant biomarkers detection. A) Schematic illustration of the biofunctionalization of graphene‐based field‐effect transistor (GFET) biosensor for cTnI detection. B) Transfer characteristics of cTnI inducer‐modified GFET for the differentiation of healthy individuals from patients with mild‐to‐moderate myocardial infarction. Reproduced with permission.^[^ ^51^ ^]^ Copyright 2022, Elsevier. C) Schematic diagram of the integrated sensor device, purified proteins/serum/plasma reservoirs (#1), washing buffer solution (#2), elution buffer (#3). A (normally‐closed) micropump (#4), waste collection chambers (W#1–4), micromixer (#7), air inlet (#6), and four FET detection chambers (FET# A–D). D) Structure of the FET detection chamber on the sensor device, where the dotted line represents a lateral view of the FET‐A chamber. The cardiovascular disease (CVD) biomarkers detection processes include D‐1) surface‐decoration of specific aptamers, D‐2) washing away of un‐immobilized aptamers, D‐3) addition of biomarkers, D‐4) capture of biomarkers by specific aptamers on the FET surface, D‐5) elution, and D‐6) re‐addition of targets for further tests. The other three chambers have similar detection processes. Reproduced with permission.^[^ ^109^ ^]^ Copyright 2019, Elsevier.

Cardiovascular diseases (CVDs) are one of the largest cause of human death.^[^ ^140^ ^]^ Among all CVDs, acute myocardial infarction (AMI) is a disease worth noting, that is, the sudden interruption of blood flow to the heart.^[^ ^141^ ^]^ Cardiac troponin I (cTnI) is recognized as one of the highly recommended biomarkers because of its extremely high sensitivity and specificity in the diagnosis of myocardial injury.^[^ ^142^ ^]^ Prajesh et al. has developed a highly sensitive biosensor for detecting cTnI by immobilizing thiolated aptamer on the surface of gate electrode through Au─S covalent bonds.^[^ ^113^ ^]^ The label‐free biosensing device exhibits a significant current response to cTnI with a concentration of 0.1 ng mL^‒1^. Further, Szunerits et al. designed an aptamer/polyethylene glycol (PEG)‐modified graphene‐based FET (GFET) to detect cTnI and differentiate between mild‐moderate‐severe myocardial injury in a clinical setting.^[^ ^51^ ^]^ Azide functionalized aptamers that are specific for cTnI were immobilized on ethynyl‐modified graphene channels by click chemistry (Figure 5A). The PEG chain was conjugated as an ionic buffer layer on the graphene device next to the cTnI aptamer enabled furthermore, which effectively reduced the Debye screening effect induced by the high ionic concentration environment and improved the sensing sensitivity of the GFET in physiological solutions. The proposed electrical sensor achieves cTnI detection at an order of magnitude lower concentration than the 40 pg mL^‒1^ cutoff for detecting myocardial injury and successfully discriminating 15 patients with mild‐moderate‐myocardial injury in less than 10 min according to changes in Dirac points (Figure 5B). The relative resistance of the PEG/Graphene FET increases linearly with the logarithm of cTnI concentration in the range of 10^−11^–5 × 10^−10^ g mL^−1^. The detection limit of this biosensor is 3.34 × 10^−12^ g mL^−1^, which is well below previously reported SiNWs or metal oxides‐based FET biosensors.^[^ ^51^, ^103^, ^105^ ^]^ *N*‐terminal propeptide of B‐type natriuretic peptide (NT‐proBNP) with a longer half‐life in the blood circulation process is also recognized as a specific biomarker for the diagnosis of heart failure. Wang and co‐workers developed an aptamer functionalized AlGaN/GaN FET (HEMT) biosensor for the rapid and highly sensitive detection of NT‐proBNP in clinical serum without sample pretreatment.^[^ ^143^ ^]^ The prepared HEMT produces a liquid capacitor in the biosensing region under high electrical field due to its unique gate electrode and channel gap design, resulting in the magnification of channel current and improvement in detection sensitivity. The high field regulation technique breaks the limitation of charge screening effect and facilitates protein detection in high salinity environment, enabling HEMT to detect NT‐proBNP with high sensitivity and selectivity in complex body fluid environment. In pursuit of higher accuracy of cardiovascular disease detection, Lee et al. integrated different aptamer‐modified FET sensor arrays into a microfluidic platform to contemporize the quantification of four CVDs biomarkers (C‐reactive protein (CRP), NT‐proBNP, cTnI, and fibrinogen) from clinical samples.^[^ ^109^ ^]^ The integrated sensing device consists of four FET detection chambers and an automated microfluidic device (Figure 5C). The thiol‐modified aptamers were distributed into the FET detection chambers through a microfluidic chip and simultaneously immobilized on the respective FETs through Au─S bonds (Figure 5D). During the detection process, the sample solution covers the sensor channel and gate to form a liquid capacitor. Binding of CVDs biomarkers to aptamers alters the solution capacitance and induces the potential drop across the dielectric layer, which in turn results in the drain current change. The capacitance‐mediated structure endows the FET sensor with “beyond Debye length” testing capability at high salt concentrations, allowing direct testing of clinical samples without the need for dilution of biological samples. Therefore, the integrated device enables rapid (5 min) analysis of four cardiovascular disease biomarkers from clinical samples (∼ 4 μL), and the detection limits for CRP, NT‐proBNP, cTnI, and fibrinogen are 1.4 × 10^−7^ g mL^−1^, 8.32 × 10^−13^ g mL^−1^, 3.94 × 10^−13^ g mL^−1^ and 2.02 × 10^−4^ g mL^−1^, respectively. This integrated portable multi‐analyte detector shows great commercialization promise for next‐generation point‐of‐care devices.

#### 4.1.3 Neurological diseases relevant biomarkers detection

**FIGURE 6:**
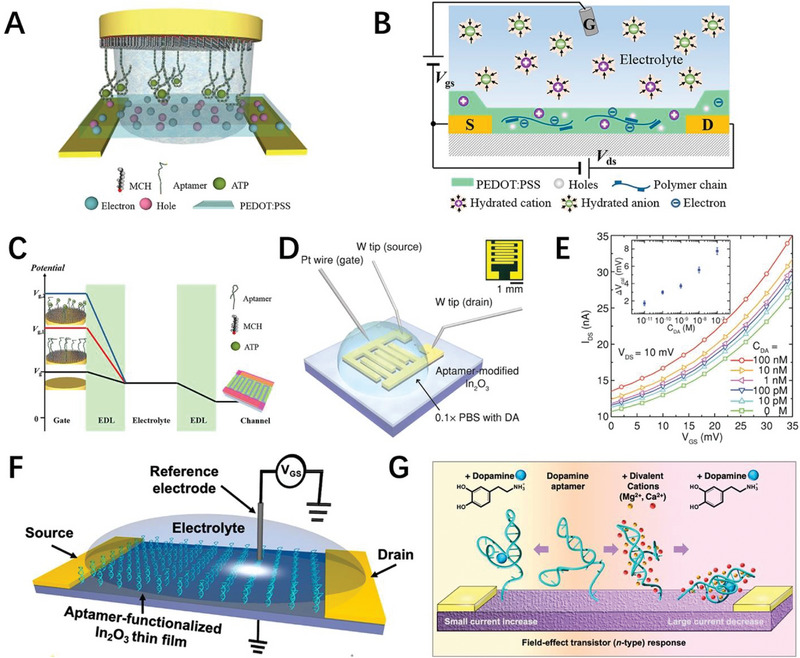
Neurological diseases relevant biomarkers detection. A) Schematic diagram of an organic electrochemical transistor (OECT) with aptamer‐functionalized gate electrode. B) De‐doping process of PEDOT: PSS channel with positive gate bias applied. C) Schematic diagram of OECT potential drops for Au electrode (*V*_g_) after incubation in aptamer solution (*V*_g, 1_), and after administration of ATP (*V*_g, 2_). Reproduced with permission.^[^ ^111^ ^]^ Copyright 2019, Elsevier. D) Schematic diagram of aptamer functionalized In_2_O_3_ biosensors for dopamine detection. E) Electrical response of aptamer functionalized In_2_O_3_ biosensors toward dopamine with different concentrations. Reproduced with permission.^[^ ^116^ ^]^ Copyright 2015, American Chemical Society. F) Schematic of a dopamine specific aptamer functionalized In_2_O_3_ field‐effect transistor (FET) biosensor. G) Effect of divalent metal cations on the conformation of aptamers upon binding to dopamine. Reproduced with permission.^[^ ^148^ ^]^ Copyright 2021, American Chemical Society.

With the aging of population, the number of patients with neurological diseases such as Parkinson's disease, Alzheimer's disease (AD) and schizophrenia continues to increase.^[^ ^144^ ^]^ Neurotransmitter is an endogenous chemical messenger that transmits signals from one nerve cell to another nerve cell through synapses.^[^ ^145^ ^]^ Various neurotransmitters and biomolecules including dopamine (DA), adenosine triphosphate (ATP), acetylcholine (Ach), epinephrine (EP), norepinephrine (NE) and nitric oxide (NO) have been used as diagnostic and prognosis biomarkers of neurological diseases.^[^ ^146^ ^]^ Mayer et al. designed an aptamer‐functionalized poly(3,4‐ethylenedioxythiophene)‐doped poly(styrenesulfonate) (PEDOT: PSS) organic electrochemical transistor (OECT) for the detection of ATP of Parkinson's disease (Figure 6A).^[^ ^111^ ^]^ The ATP‐specific aptamer was immobilized on the gate Au electrode though a thiol–gold bond. Binding of ATP to the specific aptamer induces a conformational change of the aptamer, bringing the negatively charged aptamer and ATP closer to the Au electrode and reducing the gate voltage. The decrease in gate voltage further leads to a decrease in cations that penetrate into the PEDOT: PSS channel to compensate for the pendant sulfonate anions on PSS, which enhance the hole density of the conducting polymer channel, thus resulting in an increase of source‐drain current (Figure 6B). The potentiometric sensor converts gate potential changes caused by binding events between ATP and aptamer into changes in PEDOT: PSS channel current and successfully achieves a detection limit of 10 pM benefiting from the high transconductance and amplification properties of interdigitated OECT (Figure 6C). Weiss et al. fabricated an aptamer functionalized In_2_O_3_ FET biosensor for dopamine detection (Figure 6D).^[^ ^116^ ^]^ Specifically, the thiol‐modified DNA aptamer was immobilized on In_2_O_3_ semiconductor channel with the assistance of (3‐aminopropyl)trimethoxysilane and 3‐maleimidobenzoic acid *N*‐hydroxysuccinimide ester linker molecule. The electrostatic gating effect of negatively charged DNA on the channel surface reduces the carrier concentration of the n‐type In_2_O_3_. When positively charged dopamine binds to the aptamer, the carrier concentration of the aptamer‐functionalized In_2_O_3_ FET partially recovers and the drain current increases. The FET biosensor based on aptamer functionalized ultrathin In_2_O_3_ successfully achieved the detection of dopamine—with detection limits as low as sub‐nanomolar, which is sufficient to detect dopamine in the physiological range of basal extracellular brain level (Figure 6E). In addition to changing the surface charge of the channel material by binding and detaching the aptamer to the target molecule, metal cations can also be used to redirect the aptamer configuration to change the surface charge of the channel material to achieve highly sensitive sensing.^[^ ^147^ ^]^ Nako and co‐workers used a dopamine specific aptamer that is sensitive to divalent cations to improve the detection sensitivity of dopamine (Figure 6F).^[^ ^148^ ^]^ Mg^2+^ and/or Ga^2+^ divalent cations effectively stabilize the aptamer conformations prior to exposure to dopamine. When dopamine binds to the aptamer, Mg^2+^ and/or Ca^2+^ induce the conformational rearrangement of the aptamer on the In_2_O_3_ surface to form a new secondary structural motif (parallel G‐quadruplex‐like) that enables the dopamine aptamer‐target complexes to be redirected within *λ*_D_ closer to the channel surface, resulting in more intense electron depletion and reduced current response (Figure 6G). In this case, the change in the binding signal of the aptamer to dopamine is amplified, thereby greatly improving the detection sensitivity.

#### 4.1.4 Pathogen identification

In the past few years, a lot of research work has been conducted to apply aptamer‐functionalized FET biosensors in the detection of various viruses such as SARS‐CoV‐2, human immunodeficiency virus (HIV), influenza, and so forth The following section will briefly introduce some of their applications in virus analysis and detection.

**FIGURE 7:**
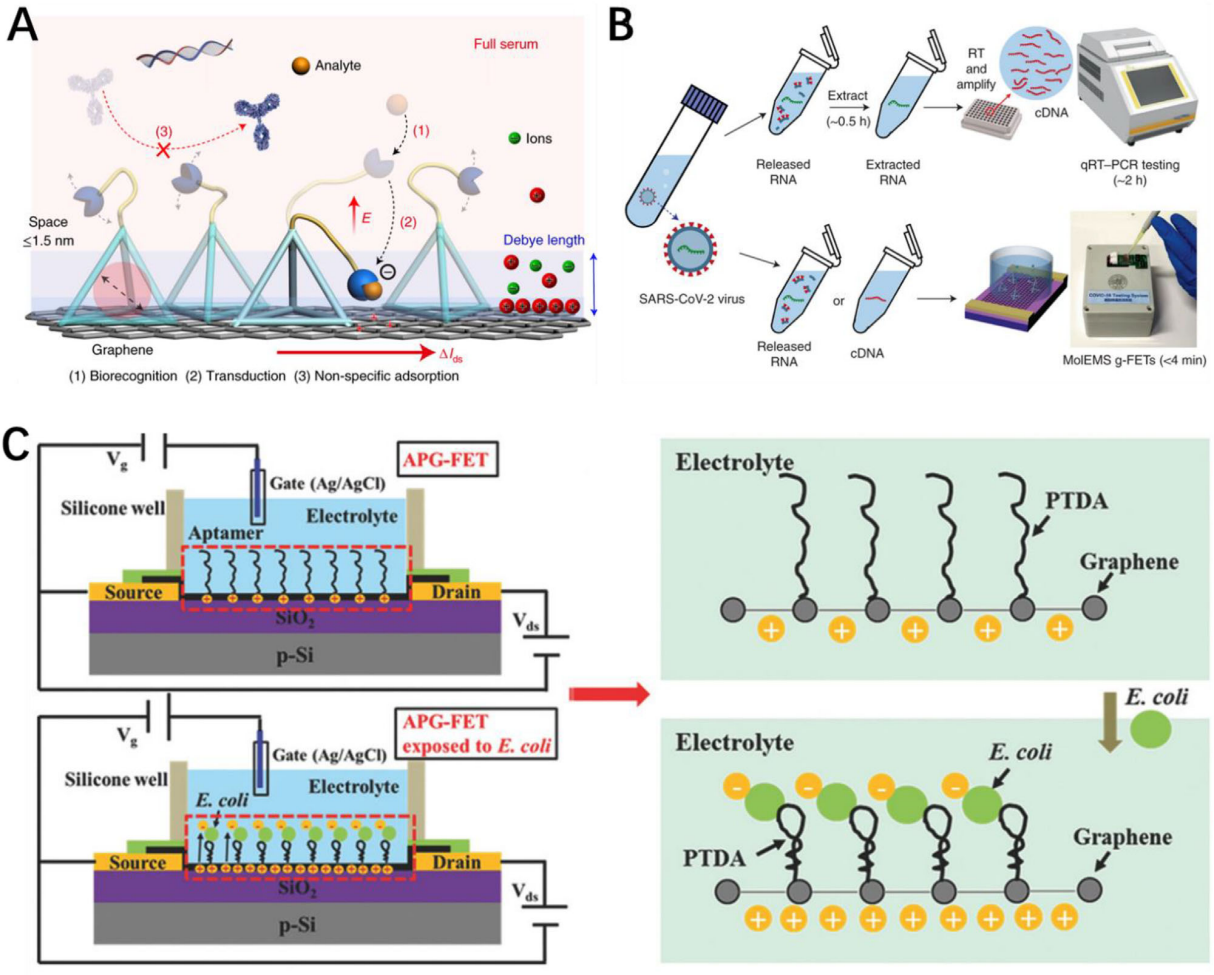
Pathogen identification. A) Sensing mechanism of MolEMS. Under the action of static electricity, the flexible cantilever pulls the recognition events to the graphene based field‐effect transistor (GFET) channel, which leads to efficient biometric recognition and signal transduction. B) Detection process of SARS‐CoV‐2 nucleic acid by qRT‐PCR and portable prototype device based on integrated MolEMS GFETs electromechanical biosensor. Reproduced with permission.^[^ ^10^ ^]^ Copyright 2022, Springer Nature. C) Sensing mechanism of aptamer‐functionalized FET biosensors is attributed to the binding of *E. coli* causes the change of aptamer structure and charge distribution in the graphene FET biosensor. Reproduced with permission.^[^ ^126^ ^]^ Copyright 2017, John Wiley & Sons.

Coronavirus disease 2019 (COVID‐19) caused by severe acute respiratory SARS‐CoV‐2 infection posing major challenges to global public health and medical services.^[^ ^149^ ^]^ The fast and high‐frequency direct detection of SARS‐CoV‐2 nucleic acid is essential to control the new coronary pneumonia epidemic. Wang et al. report an aptamer functionalized microelectromechanical system (MolMES) immobilized on a liquid‐gated graphene FET for rapid and ultrasensitive detection of unamplified SARS‐CoV‐2 nucleic acids in biological fluids.^[^ ^10^ ^]^ The MolMES consists of a stiff tetrahedral base with six ds‐DNA edges linked by single‐stranded hinges and a flexible cantilever composed of an ssDNA (Figure 7A). The entire MolMES is covalently immobilized on the graphene FET through amide bonds. The other end of the flexible arm ssDNA is equipped with the SARS‐CoV‐2 RNA aptamer of the material to be detected. When negative gate voltage is applied to the liquid electrolyte, the local electric field will actuate the flexible cantilever bound to SARS‐CoV‐2 RNA downwards, and drive the cantilever closer to the Debye radius of GFET to greatly improve the sensing performance. The electromechanical biosensor was further installed into an integrated portable prototype device for on‐site and point‐of‐care detection (Figure 7B). The device successfully detected SARS‐CoV‐2 RNA in all nasopharyngeal samples from 33 COVID‐19 patients in less than 4 min, the detection limit reaches as low as ∼ 0.02 copies μL^‒1^.

Avian influenza viruses (AIV) such as H5N1 are highly pathogenic, and their early detection is essential to prevent and control global influenza outbreaks.^[^ ^150^ ^]^ Ahn et al. reported a label‐free aptamer functionalized FET biosensor for the detection of AIV in chicken serum.^[^ ^123^ ^]^ The aptamer was fixed on a gold electrode via a thiol linker, and the specific binding between target protein and gold electrode will cause the variation of surface potential, thereby generating a current signal response. The aptamer modified FET biosensor shows a linear range of 10 pM–10 nM, and the detection limit reaches as low as 5.9 pM. The device shows the characteristics of simple and low‐cost architecture, portability, and high sensitivity, and it is expected to be used for instant diagnosis of H5N1 AIV in clinical samples.

Acquired immune deficiency syndrome (AIDS) is an infectious disease caused by HIV. Early diagnosis and targeted treatment are essential to control HIV and prevent AIDS.^[^ ^151^ ^]^ Ruslinda et al. developed an aptamer functionalized diamond FET biosensor for applications in HIV detection.^[^ ^152^ ^]^ The diamond surface was aminated by UV light irradiation in an ammonia atmosphere. After partial amination of the diamond surface, the RNA^Tat^ aptamer was immobilized on the diamond surface via a terephthalic acid linker molecule. The conjugation of HIV‐1 Tat protein and aptamer generates a significant gate potential change and thus an improved detection sensitivity.

*Escherichia coli (E. coli)* is an opportunistic pathogen that can cause diarrhea, hemorrhagic colitis, cystitis, cholecystitis, and even sepsis.^[^ ^153^ ^]^ Aptamer modified FET biosensors have been comprehensively used to detect *E. coli* with high sensitivity and fast response speed.^[^ ^154^ ^]^ Wu et al. used pyrene phosphor amidite to immobilize aptamer on the surface of graphene to obtain a functionalized FET biosensor for highly sensitive detection of *E. coli* (Figure 7C).^[^ ^126^ ^]^ The detection mechanism of the FET biosensor is based on conformational change of aptamer caused by the specific binding of *E. coli* to aptamer. The change in the conformation of aptamer makes the negatively charged *E. coli* close to the graphene surface, which effectively increases the hole density of the sensing channel and thus the response current. The detection limit of the *E. coli* specific aptamer functionalized FET biosensor reaches as low as 10^2^ CFU mL^‒1^. The high sensitivity and selectivity FET biosensors open up possibilities for the detection of pathogens in biological environment.

### 4.2 Application in environmental monitoring

In the past few years, aptamer functionalized FET biosensor has been successfully employed as an efficient detection platform in environmental monitoring due to its low detection limit, excellent selectivity, and portable operation potential (Table 2).

**TABLE 2:** Representative summary of aptamer functionalized FET biosensors toward environmental monitoring.

Aptamer functionalized FET biosensors	Types of pollution	Pollutant	Linear range (M)	Detection limit (M)	Ref.
Multichannel carbon nanofibers FET	Antibiotic	Bisphenol A	10^−15^–10^−11^	10^−15^	[74]
CPPyNPs FET	Antibiotic	Bisphenol A	10^−15^–10^−11^	10^−15^	[83b]
rGO FET	Antibiotic	Tobramycin	10^−8^–10^−4^	3 × 10^−10^	[155]
ZnO FET	Antibiotic	Tobramycin	10^−9^–10^−7^	10^−10^	[156]
MoS_2_ FET	Antibiotic	Kanamycin	10^−9^–10^−4^	1.06 × 10^−9^	[157]
HfO_2_/GFET	Antibiotic	Kanamycin A	< 10^−7^	1.15 × 10^−8^	[158]
GFET	Heavy metal ions	Hg(II)	5 × 10^−20^–2.5 × 10^−10^	5 × 10^−20^	[10]
GFET	Heavy metal ions	Hg(II)	10^−10^–10^−7^	4 × 10^−11^	[80]
GFET	Heavy metal ions	Hg(II)	5 × 10^−9^–2 × 10^−7^	5 × 10^−9^	[85c]
GO/Al_2_O_3_ FET	Heavy metal ions	Hg(II)	1.25–12.5 × 10^−6^	1.2 × 10^−6^	[159]
rGO‐COOH FET	Heavy metal ions	Pb(II)	5 × 10^−9^–5 × 10^−8^	5 × 10^−12^	[160]
SWNT FET	Heavy metal ions	Pb(II)	5 × 10^−12^–5 × 10^−7^	2 × 10^−12^	[161]
GFET	Heavy metal ions	Cu(II)	10^−8^–3 × 10^−6^	10^−9^	[162]
SiNW FET	Heavy metal ions	UO_2_(II)	10^−12^–10^−8^	10^−12^	[163]

Abbreviations: FET, field‐effect transistor; GFET, graphene‐based FET; SiNW, silicon nanowire.

#### 4.2.1 Antibiotic detection

**FIGURE 8:**
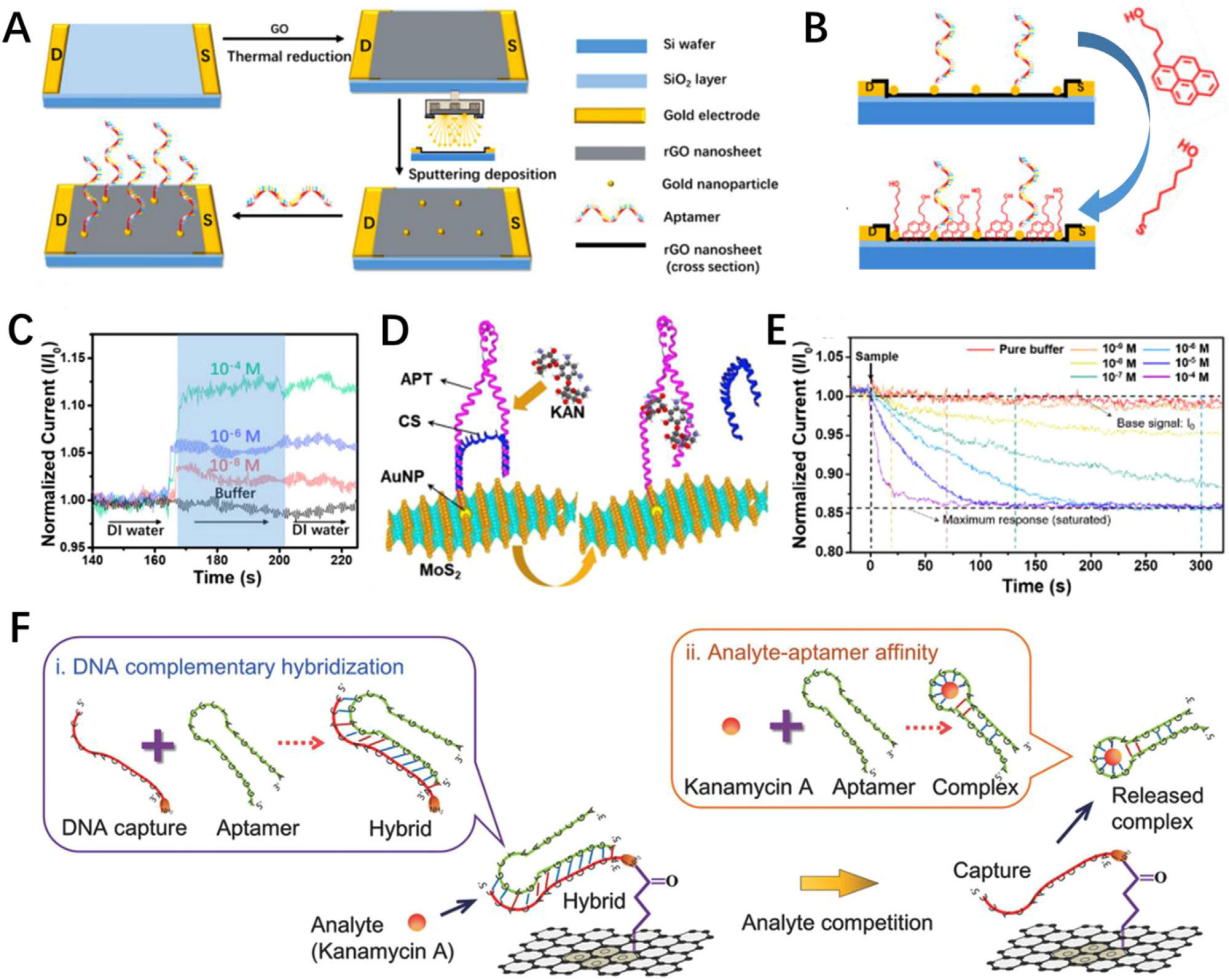
Antibiotic detection. Schematic diagram of A) the rGO/aptamer field‐effect transistor (FET) biosensor fabrication process and B) the chemical blocking steps. C) Real‐time recording of current response of GFET biosensors toward tobramycin with concentrations ranging from 10^−8^–10^−4^ M. Reproduced with permission.^[^ ^155^ ^]^ Copyright 2019, Elsevier. D) Schematic diagram of the MoS_2_/APT/CS FET biosensor structure and the sensing mechanism. APT: an aptamer composed of a DNA complementary strand (CS). E) Time‐resolved normalized current responses of MoS_2_/APT/CS FET biosensor toward kanamycin (KAN) with concentrations ranging from 10^−9^ to 10^−4^ M. Reproduced with permission.^[^ ^157^ ^]^ Copyright 2017, Elsevier. F) Competitive affinity sensing principle of graphene FET biosensor for the detection of KAN A. Reproduced with permission.^[^ ^158^ ^]^ Copyright 2016, John Wiley & Sons.

Antibiotics are one of the important medical discoveries and have played an important role in the treatment of human diseases, livestock, and aquaculture.^[^ ^164^ ^]^ However, the abuse of antibiotics has led to the emergence of resistant genes and resistant bacteria, posing a huge threat to the ecological environment and human health.^[^ ^165^ ^]^ In order to effectively control the pollution of antibiotics, it is necessary to monitor the antibiotics in real time. Mao et al. designed an aptamer modified rGO FET biosensor to achieve highly sensitive detection of tobramycin (Figure 8A).^[^ ^155^ ^]^ A novel chemical passivation layer was modified on the channel surface based on blocking strategy to eliminate non‐specific adsorption and improve the selectivity of the rGO FET biosensor (Figure 8B). The rGO/aptamer/passivation layer biosensor exhibits a fast (1–5s) and sensitive response to tobramycin with a detection limit as low as 0.3 nM (Figure 8C). Meanwhile, they also designed a kanamycin aptamer (APT) modified MoS_2_ FET biosensor for the highly specific detection of kanamycin (KAN) (Figure 8D).^[^ ^157^ ^]^ The APT was stabilized on the surface of MoS_2_ with gold nanoparticles as linkers, and short complementary strand DNA (CS) was linked to APT through base complementary pairing to fold into a conjugated structure. The sensing principle relies on the addition of KAN to uncoil APT/CS pairing and form a complex APT/KAN tertiary structure. When the negatively charged CS is released into the bulk solution, the reduction of the negative charge within the Debye length of the MoS_2_ surface leads to a decrease in p‐doping on MoS_2_ and reduces the source‐drain current (Figure 8E). The sensing mechanism based on charge release from probe effectively overcomes the limitation of Debye screening and improves the sensitivity of the device. The sensitivity and LOD of MoS_2_/APT/CS biosensor for KAN are 1.85–4.43 M^‒1^ and 1.06–0.66 nM. Wang et al. designed an aptamer functionalized high‐κ solid gate graphene FET biosensor based on the principle of aptamer‐competitive affinity principle for the detection of kanamycin A (Figure 8F).^[^ ^158^ ^]^ DNA anchor is immobilized on the surface of the graphene channel through 1‐pyrenebutyric acid *N*‐hydroxysuccinimide ester (PBA‐NHS) linker, and aptamer is then connected to the DNA anchor through base complementary pairing to complete the aptamer functionalization. The sensing mechanism of kanamycin A specific aptamer functionalized graphene FET biosensor is based on the competitive affinity of kanamycin A. Specifically, kanamycin A competitively binds to the aptamer and folds into a hairpin complex, which breaks the DNA anchor‐aptamer hybridization. As a result, the negatively charged hairpin complexes detach from the graphene surface, reducing the hole density of graphene channel and *I*_ds_ of the biosensor. The aptamer functionalized GFET biosensor achieved high sensitivity detection of kanamycin A with a detection limit of 11.5 × 10^−9^ M.

#### 4.2.2 Metal ions detection

Heavy metal ions would interact strongly with proteins and cause protein inactivation, which seriously influences body metabolism and threatens human health.^[^ ^166^ ^]^ The detection of heavy metal ions in ecological environment is of vital importance to reduce heavy metal pollution and has attracted increasing attention worldwide.^[^ ^167^ ^]^

Pb(II) ions are highly toxic and bioaccumulative that can cause serious damage to the human nervous system, brain, and kidneys even at low concentrations.^[^ ^168^ ^]^ Liu et al. have developed a reusable biosensor for Pb(II) ions detection by immobilizing duplex DNA that is composed of a single‐stranded DNA (CS‐DNA) and a G‐quadruplex (G4) aptamer to single‐walled CNTs.^[^ ^161^ ^]^ The CS‐DNA with the terminal amino group was covalently conjugated on the surface of single‐walled CNTs through PBASE linker, and the G4 aptamer was then connected to the CS‐DNA through base complementary pairing. During the detection, Pb(II) drives the unwinding of CS‐DNA and G4 aptamer to form a stable G4/Pb(II) complex. The despiralization of the DNA duplex reduces the resistance of the single‐walled CNTs and improves the signal output of the device. The relative resistance of the biosensor increases linearly with the logarithm of Pb(II) concentration in the range 1 ng L^‒1^–100 μg L^‒1^. The detection limit of this biosensor is 0.39 ng L^‒1^, which is below the standard of the World Health Organization (0.01 mg L^‒1^). In addition, CS‐DNA could recombine with G4 aptamer after each detection based on the dynamic reversible behavior of base complementary pairing, which greatly improves the reusability of the biosensor.

Arsenic (As), especially As(III) pollution in drinking water is an important water pollution problem that needs to be solved urgently.^[^ ^169^ ^]^ The CPPy‐encapsulated CFMNSs were immobilized on the amination interdigitated microelectrode array (IDA) through the condensation reaction, and the amino‐modified aptamer is then covalently bound to CFMNSs surface. The As(III) can convert single‐stranded DNA into folded and condensed structures through strong hydrogen bonding with amine groups of aptamers, resulting in the increase of negative charges in the liquid ionic medium near the surface of CFMNS. The negative charge causes the accumulation of hole carriers in the CFMNS and increases the current. The aptamer functionalized CFMNS FET biosensor exhibits high specificity, and it could identify As(III) from the river water successfully. In addition to high selectivity, the biosensor also shows extraordinary response performance with a response time of less than 1 s. The design of aptamer functionalized CFMNS FET biosensor provides a general strategy to construct a variety of detection tools to satisfy a variety of practical applications.

## 5 CONCLUSION AND PERSPECTIVES

This review has focused on the research progress in the development and application of aptamer functionalized FET biosensors in disease diagnosis and environmental monitoring. The sensing mechanism and typical 1D and 2D channel materials used in aptamer functionalized FET biosensors were summarized and highlighted. The non‐covalent and covalent aptamer functionalization strategies and applications of aptamer functionalized FET biosensors in disease diagnostics and environmental monitoring were comprehensively reviewed and discussed. This review will provide theoretical guidance for the design and development of aptamer functionalized FET biosensors with high sensitivity and selectivity, and offers technical support for the development of efficient disease diagnostic technology and bioanalytical equipment in the future.

The past decade has witnessed tremendous growth in the development and application of aptamer functionalized FET biosensors, but the currently developed aptamer‐functionalized FET devices still suffer from the limitations such as relatively poor reproducibility and high cost. The improvement of device reproducibility depends on controlling the growth or preparation methods of channel materials to obtain large‐scale channel materials with uniform performance and structure. At the same time, creating a source of reproducible high affinity aptamers, simplifying and regulating the aptamer functionalization process could help reduce product‐to‐product variation and improve device reproducibility. The reason is that the binding affinity of aptamer would be diminished by the interference molecules or ions in real samples with particularly complex environment. The recognition ability of aptamer upon attaching on a substrate would be further weakened. Also, it takes a long time (10 ∼ 15 min) for the achievement of effective target binding. The evaluation of aptamer binding capability is a challenging task that non‐aptamer sequences might show false positive binding results without appropriate controls. There remain many shortcomings preventing aptamers’ practical application. The traditional SELEX technique for selecting aptamer is time‐consuming, labor intensive, and expensive. Most of the reported aptamers were applied in in vitro research, and the in vivo behaviors of aptamers may be complicated and undefined. Furthermore, exploring innovative directional surface functionalization methods to minimize the non‐specific adsorption of interfering molecules on the surface of channel materials is also an important factor to improve device reproducibility. Improvements in micro‐nano fabrication technology may offer attractive alternatives to reduce device production cost. Further, the development prospect of aptamer‐functionalized FET biosensors relies on combining high‐stability and low‐cost sensing devices with signal conversion and transmission units, and develops portable digital monitoring devices with the assistance of smart phones and Bluetooth signal transmission units. The development of portable biosensors is expected to get rid of the regional restrictions on large‐scale testing instruments. Moreover, the intelligent mobile platform endows the data with the characteristics of high spatial and temporal resolution, which contributes to the achievement of real‐time and timely medical care, precision treatment, and personalized management.

## CONFLICT OF INTEREST STATEMENT

The authors declare no conflicts of interest.
